# Myocardial Infarction Signs and Symptoms: Females vs. Males

**DOI:** 10.7759/cureus.37522

**Published:** 2023-04-13

**Authors:** Kyle J Schulte, Harvey N Mayrovitz

**Affiliations:** 1 Medicine, Nova Southeastern University Dr. Kiran C. Patel College of Allopathic Medicine, Fort Lauderdale, USA; 2 Medical Education, Nova Southeastern University Dr. Kiran C. Patel College of Allopathic Medicine, Fort Lauderdale, USA

**Keywords:** women, men, male, female, pathophysiology, symptoms, heart attack, risk-factors, sex differences, myocardial infarction

## Abstract

Cardiovascular disease is the number one killer of females in the United States today, and myocardial infarction (MI) plays a role in many of these deaths. Females also present with more “atypical” symptoms than males and appear to have differences in pathophysiology underlying their MIs. Despite both differences in symptomology and pathophysiology being present in females versus males, a possible link between the two has not been studied extensively. In this systematic review, we analyzed studies examining differences in symptoms and pathophysiology of MI in females and males and evaluated possible links between the two. A search was performed for sex differences in MI in the databases PubMed, CINAHL (Cumulative Index to Nursing and Allied Health Literature) Complete, Biomedical Reference Collection: Comprehensive, Jisc Library Hub Discover, and Web of Science. Seventy-four articles were ultimately included in this systematic review. Typical symptoms for both ST-elevation myocardial infarction (STEMI) and non-STEMI (NSTEMI) such as chest, arm, or jaw pain were more common in both sexes, but females presented on average with more atypical symptoms such as nausea, vomiting, and shortness of breath. Females with MI also presented with more prodromal symptoms such as fatigue in days leading up to MI, had longer delays in presentation to the hospital after symptom onset, and were older with more comorbidities than males. Males on the other hand were more likely to have a silent or unrecognized MI, which concurs with their overall higher rate of MI. As they age, females have a decrease in antioxidative metabolites and worsened cardiac autonomic function than male. In addition, at all ages, females have less atherosclerotic burden than mles, have higher rates of MI not related to plaque rupture or erosion, and have increased microvasculature resistance when they have an MI. It has been proposed that this physiological difference is etiologic for the male-female difference in symptoms, but this has not been studied directly and is a promising area of future research. It is also possible that differences in pain tolerance between males and females may play a role in differing symptom recognition, but this has only been studied one time where females with higher pain thresholds were more likely to have unrecognized MI. Again, this is a promising area for future study for the early detection of MI. Finally, differences in symptoms for patients with different atherosclerotic burden and for patients with MI due to a cause other than plaque rupture or erosion has not been studied and are both promising avenues to improve detection and patient care in the future.

## Introduction and background

As of this writing, cardiovascular disease is the number one killer of females in the United States, having been responsible for 301,280 deaths in 2019 [1-3]. Considering just myocardial infarction (MI), males have a higher incidence than females, with males accounting for approximately 70% of MIs and having an MI 7-10 years earlier than females [2,4]. Despite this, females experience a greater one-year mortality rate after an MI with an odds ratio (OR) of 1.6 [5]. Some of this mortality difference may be attributable to differences in age and comorbidity burden at first MI, where females present later and with more risk factors such as type 2 diabetes mellitus [1,5-7]. Despite the impact of MI on females, it is less well defined than for males, with the symptoms of females described as “atypical” versus the “typical” male symptoms [8,9].

A part of the greater mortality burden in females may be due to the lack of recognition of these atypical symptoms by physicians and patients alike, as females with MI are less likely to receive timely and evidence-based interventions upon MI symptom onset [5,7,10]. Females who are having an MI also tend to present to the hospital later after symptom onset than males, which may indicate a possible lack of knowledge in the general population of the dangers of MI in females or a lack of knowledge of the differences in symptomology that females present with [11-13]. These differences appear to be present across race and culture, though Black and Hispanic females in the United States are more likely to present with atypical MI symptoms than White females [14].

While some differences in treatment and in outcomes experienced by females with an MI may be due to a lack of awareness or other systemic social factors, a difference in pathophysiology leading to differences in symptoms may also be at play. Females do appear to have differing cardiac physiology and MI pathophysiology from males, which may further exacerbate the mortality difference between sexes [5,15-17]. Despite some differences in symptomology and pathophysiology having been previously considered [5,18,19], few links between the two have been established. The goal of the present investigation is to elucidate the sex differences in MI symptomology and explore the pathophysiological differences that may underlie them. 

## Review

**Figure 1:**
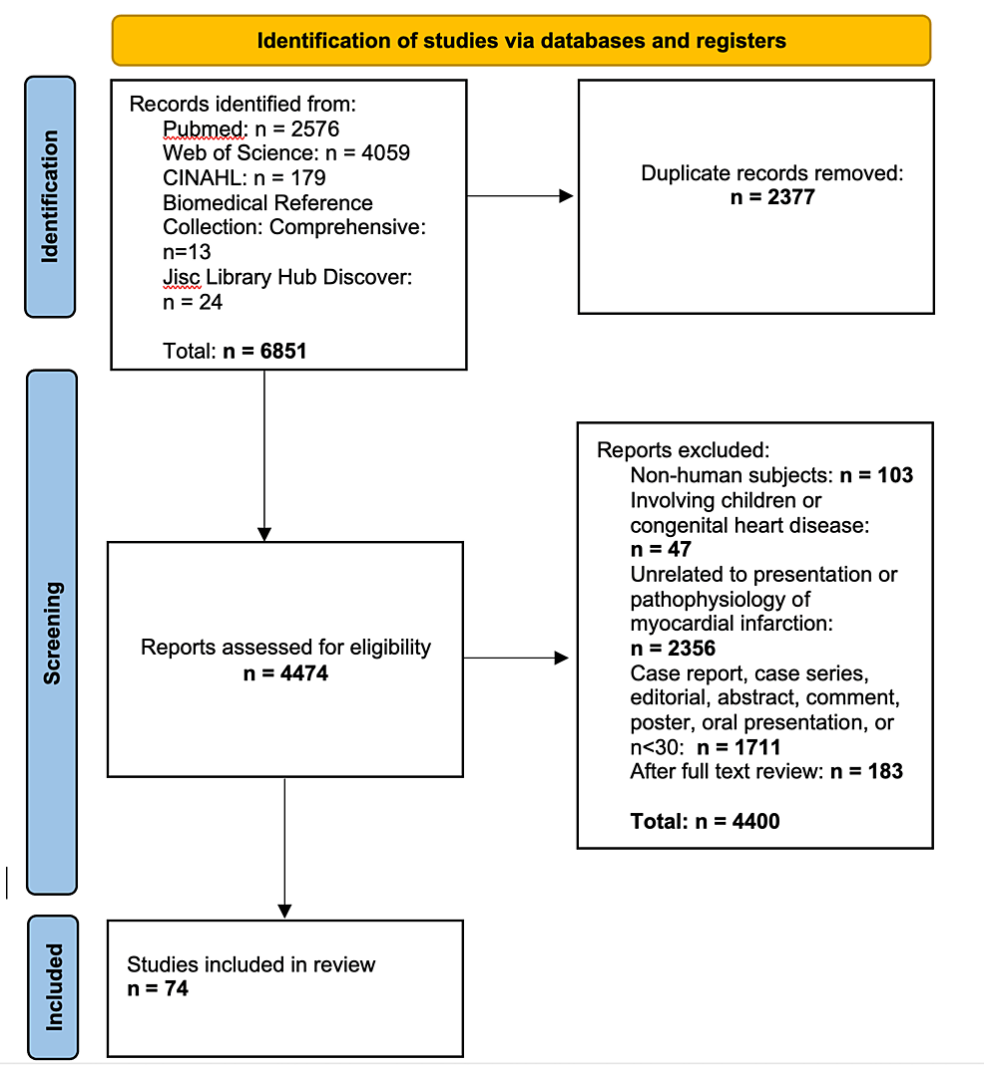
PRISMA flow chart for publication selection PRISMA: Preferred Reporting Items for Systematic Reviews and Meta-Analyses

This systematic review was created in accordance with Preferred Reporting Items for Systematic Reviews and Meta-Analyses (PRISMA) 2020 guidelines (Figure 1). Articles were included if they satisfied the following criteria: they were written in English, the study included adults with a sample size of at least 30, the study focused on MI symptomology or pathophysiology, and had a consideration of sex differences in cardiac physiology. Articles were excluded if they did not characterize symptoms at the onset of MI and had a primary outcome other than myocardial infarction, ischemic heart disease, or sex differences in cardiac physiology. PubMed, CINAHL (Cumulative Index to Nursing and Allied Health Literature) Complete, Biomedical Reference Collection: Comprehensive Edition, Jisc Library Hub Discover, and Web of Science were searched for articles satisfying the inclusion and exclusion criteria. A total of 6851 titles were retrieved, which after removing duplicates resulted in 4474 unique articles. Articles were reviewed for inclusion by one reviewer working independently. On full-text review, articles were excluded or included based on primary outcome describing symptomology or pathophysiology of MI with a focus on sex differences. In the final review, 74 articles were included. For examination of bias within included articles, both reviewers examined the full text of the articles selected.

Prevalence of typical and atypical symptoms in males vs. females

Females often present with more “atypical” symptoms than males [20-22]. Typical symptoms of both sexes are chest, arm, or jaw pain of a dull, heavy, tight, or crushing quality whereas atypical symptoms are other common but less frequent presentations such as nausea, vomiting, diaphoresis, shortness of breath, dizziness, and pain in locations other than those described earlier [8,23]. Upon arrival at hospital, both sexes report chest pain and feelings of chest tightness or pressure as their most common MI symptom [9,22,24]; however, males report chest pain 13-15% more frequently than females as their chief complaint [25,26]. One study suggests that some of the reporting differences may be due to the patient interview process associated with the clinician interview strategy [27]. 

When accounting for interview styles, males and females were equally likely to report chest pain on open-ended questioning, but males reported chest pain more frequently on narrowed questioning followed by a checklist [27]. Some symptoms are reported far more frequently by females and some physical findings are more prevalent. Symptoms reported more often by females include nausea, vomiting, dizziness, and fear of death. More frequent physical findings for females are those consistent with congestive heart failure such as lung crackles or rales [26], and the presence of dyspnea [9,24,25], although one study indicates no sex-related difference [28]. 

The location of the pain reported by females is more often the jaw or neck with other pain locations being the upper back, left arm, left shoulder, left hand, and abdomen, in no particular order of frequency [9,22,24-26,28]. With increased age, females report less chest pain and more shortness of breath although no such association was seen with males [25]. Males appear to present with more chest pain but also present with more burning or pricking pain sensation and with more diaphoresis than females [9,22]. In addition to having a wider variety of possible symptoms, females also present with more symptoms during a given MI than males [20,21,24]. For example, among patients aged 18 to 55 years, females presented with 10% more symptoms than males per MI [21], and among a patient population with average age over 75, females presented with 17% more symptoms than males per MI [20]. Finally, when analyzing for possible common symptom phenotypes (e.g. chest pain with dyspnea or chest pain with nausea and vomiting), it was reported that for females 18-55 years and over 75, there is a more heterogeneous population of symptom presentations when compared to males. Females had significantly more phenotypes and these phenotypes were more broadly distributed across the population [20,21].

Prodromal symptoms in the sexes and racial similarities

In addition to altered symptomology in the acute phase of MI, females are also more likely to have prodromal symptoms in the days and weeks leading up to an MI, with some occurring more than a year prior. The most reported of these, in the order of prevalence, are feeling tired or with unusual fatigue, sleep disturbance, anxiety, shortness of breath, and arm, back, or chest pain [15,29,30]. These prodromal symptoms have not been shown to be associated with hypertension, hyperlipidemia, or age over 50 [31], although these symptoms are also associated with some cardiovascular risk factors, including family history of cardiovascular disease, obesity, DM, prior hysterectomy, smoking, secondhand smoke exposure, and lack of regular exercise.

With respect to prodromal symptoms, over 50% of females had a disturbance in sleep from normal within four weeks of their MI versus 32% of males who experienced such sleep disturbances [29,32]. It has also been reported that sleep disturbance prior to MI may be increasing since it has been reported that more females and males experienced sleep disturbances within four weeks of MI in 2008 than in 2000 [32]. Considering the sleep issue, it should be noted that most sleep-related findings come from one laboratory [14,15,29-31,33]. This group has indicated that sleep disturbances may also be an important factor for those with other cardiovascular risk factors for those patients that have sleep disturbance accompanied by changes in thinking/remembering and increased anxiety, fatigue, and pain prior to their MI [29].

An investigation into the prevalence and extent of prodromal symptoms by age has suggested three clusters. Cluster 1 includes older patients in whom the prevalence of prodromal symptoms is limited. Cluster 2 includes a diverse group who present with feelings of unusual fatigue and sleep disturbance in over 70% of members. Cluster 3 includes the youngest patients and has more minorities than the other groups. Patients in this cluster presented with unusual fatigue, sleep disturbance, anxiety, shortness of breath, arms that felt weak or heavy, and hand and arm tingling in over 70% of members [15]. Prodromal symptoms appear to vary slightly in intensity and number by race among females [14]. In all females, at least five prodromal symptoms per MI were present with Black females more likely to experience more prodromal symptoms (7.48 per MI) and more severe prodromal symptoms than Hispanic females (6.98 per MI), who in turn were more likely to experience more prodromal symptoms and more severe prodromal symptoms than White females (5.84 per MI) [14]. It has been reported that certain prodromal symptoms are predictive of future MI for males and females, with jaw or tooth discomfort (OR 2.15), unusual fatigue (OR 2.11), arm discomfort (OR 2.00), and ache in arms (OR 1.93) being significantly predictive of an MI within 90 days [33].

Risk factors, age, and comorbidities as they relate to symptoms and presentation

Males and females have different comorbidities and risk factors when presenting with MI. On average, females having an MI are older and more likely to have a history of congestive heart failure, DM, hypertension, lower BMI, and lower smoking rates than males [6,10,22,26,28,34]. On the other hand, males are more likely to present with a history of MI and peptic ulcer disease [26]. There is conflicting evidence on whether hyperlipidemia in MI differs between the sexes, as some studies show more hyperlipidemia in females [10,22], some show greater rates in males [34], and others show no difference between the sexes [9]. It is also unclear whether a history of diagnosed angina as a risk factor differs between the sexes since one study found a greater rate of angina in females [28] and another found a greater rate of angina in males [26]. It is unclear whether some risk factors influence symptom presentation and patient interpretation of their symptoms as MI. Atypical symptoms are more common in older males and females [35,36]. However, in females with DM, one study showed no difference in atypical symptomology [35] while DM was predictive of upcoming atypical MI in a Chinese cohort. This cohort also showed female sex, previous acute MI, and hyperlipidemia as predictive factors for upcoming MI with atypical symptoms [36].

Risk factors may also be different based on patients' age. Compared to the general population, females under the age of 55 are reported to be more likely to present with an MI if they have a history of obesity, stroke, transient ischemic attack, chronic kidney disease, chronic lung disease, DM, or hypertension [37,38]. In these females, DM and hypertension account for a sixfold and threefold increase in the risk of MI, respectively [37]. In males under age 55, previous history of cardiac arrhythmia, hyperlipidemia, ST-segment elevation MI (STEMI), and coronary artery stenosis greater than 50% was found more often than in females [38].

Smoking ≥ 20 cigarettes/day was associated with an increased rate of MI in both sexes, but females smoking ≥ 20 cigarettes/day had a higher risk of MI (hazard ratio (HR) = 3.46) than males smoking ≥ 20 cigarettes/day (HR = 2.23) [4]. For persons with hypertension, the rate of MI was increased in both sexes but females had a higher HR (2.52) than males (1.71). An increased risk was also associated with DM although there were differences between type 1 and type 2 DM. Females with type 1 DM had a significantly higher risk of MI (HR = 8.18) than males with type 1 DM (HR = 2.81), and females with type 2 DM had a higher risk (HR = 1.96) than males with type 2 DM (HR = 1.33). The absolute difference in risks between the sexes did not change with age, though the relative risks compared to the general population decreased with age for both females and males [4]. One study had differing results in young patients, where females and males under 55 who had MIs had no difference in the prevalence of DM but females who had MIs and were over 55 had higher rates of DM than males [6]. 

Delays in presentation

Females tend to present to the hospital later than males after MI symptom onset [13,39,40]. Females wait to call emergency medical service (EMS) three minutes longer than males, and as measured by the EMS call, arrive at the hospital 10 minutes later than males [41]. These delays may be in part due to females being more likely to report vomiting as a chief symptom when calling EMS thereby being less likely to receive a high-priority ambulance response [23].

Ambulance-related delays are particularly present for African American females, who present to the hospital on average one hour later than the general population of males and females and 1.5 hours later than White females. For these African American females, a greater chest pain intensity was associated with an even greater delay whereas there was no delay for increased pain in White females [39]. /females who have a STEMI have a longer symptom-to-hospital arrival time than males although this time interval is not different between males and females when there is a non-STEMI (NSTEMI) [42].

There is some lack of clarity in this hospital delay in females compared to males, which may be related to typical versus atypical presentations of MI. In a Chinese cohort, females of all ages presented later than males of all ages, but when controlling for symptom type, no difference in hospital delay was seen between females and males [40]. On the other hand, a Swedish cohort showed no delay in symptom-to-hospital arrival time between males and females in the general population but for patients older than 65, the delay for 30.5% of females was more than four hours whereas only 25% of males were delayed by a similar amount [43]. Other common risk factors for symptom-to-hospital arrival time delay include being a non-White patient, low socioeconomic status, low education level, increasing age, and medical history of hypertension, DM, and renal insufficiency [13,40]. Disparities in the time delays may be related to the presence of typical versus atypical symptoms. Symptoms that match those expected for an MI reduced delay times in both sexes [12], while the presence of atypical symptoms increased delay times in both sexes with a larger delay in females than males [11].

Unrecognized and silent MI

With any discussion of symptoms of MI, it is important to discuss MIs that are asymptomatic (silent MI) and those that go unnoticed by patients and physicians (unrecognized MI). It is difficult to quantify the difference between silent MI and unrecognized MI, as all silent MIs are unrecognized by definition, while patients may vaguely recall chest pain years ago as a possible unrecognized MI, so the terms are often used interchangeably.

As with MI as a whole, males are more likely to have had a previous silent MI [44], with one study showing a rate of unrecognized MI in males ages 18-80 of 2.67 per 1000 person-years and a rate in females of 1.69 per 1000 person-years [45]. The rate of silent MI also changes with race, with Asians being most likely to have a silent MI, followed by Whites, then Hispanics, and finally African Americans [44]. Unrecognized MI accounts for approximately 30% of MIs in females and 16% of MIs in males [45], and somewhere between 20% and 40% of all MIs in the general population [46]. Unrecognized MI is also associated with hypertension (OR 1.82), being a former or active smoker (OR 1.82), and elevated blood glucose (OR 1.41). The entire spectrum of blood glucose dysfunction appears to be related to silent and unrecognized MI and may affect males and females differently. Impaired glucose tolerance is more common among females (OR 4.1) with unrecognized MI but not associated with unrecognized MI in males [47].

On the other hand, impaired fasting glucose and pre-diabetes have been shown to increase the risk of unrecognized MI in males but not females [48]. Patients with type 2 DM are more likely to have had silent or unrecognized MI regardless of sex, with an increased risk in females with microalbuminuria [49,50]. The prognosis of unrecognized MI is most likely similar to recognized MI but difficult detection makes mortality estimation difficult. MRI is significantly more accurate at detecting silent MI than ECG or echocardiography and its use in the future may help better quantify and detect silent MI in the general population [46].

Pathophysiology

There are physiological and pathophysiological differences in females and males that may impact differences in MI aspects between the sexes, but direct connections are not fully elucidated. For example, as females age, there is increased acetylation of mitochondrial DNA indicating decreased mitochondrial function compared to males. Young females have increased antioxidative metabolite presence when compared to males, but this difference is lost with age. There is also increased inflammatory macrophage presence and a corresponding increase in pro-inflammatory molecules such as NF-kB and IL-18 in female hearts where no increase is seen in males [17]. Females also show decreased heart rate variability with age as compared to males, suggesting a greater age-related reduction in autonomic function in females versus males [51]. It has also been reported that the presence of angiotensin I converting enzyme phenotype DD appears to double the risk of MI in males but not in females [52]. Though females present with a differing androgen hormonal profile than males, no association between differing endothelial or cardiac androgen receptor subtypes and MI or other cardiovascular diseases has been shown [53].

The role of atherosclerotic burden

At younger ages, females have an overall lower atherosclerotic burden as measured by intima media thickness than males, though this difference attenuates with age and is nonsignificant after age 65 [54]. When measured directly, males also have an increased number of atherosclerotic plaques on average compared to females at all ages [54-56]. One study showed no increase in plaque burden in females with age [55], though others found an increase but less than the increase seen in males [54,56]. When females have a >75% stenosis as measured by the diameter of plaque compared to the diameter of the coronary artery, there is a five-to-seven-fold increase in risk for infarction, while males show a doubling in risk [55,56]. When females have a low obstructive plaque burden but still have an MI, there is evidence that most of these MIs are due to plaque rupture or erosion, though this study did not examine MIs of males with the same conditions [57].

Coronary perfusion differences between males and females also impact MI feature differences. About 5-10% of MIs are considered to occur with nonobstructive coronary artery disease (myocardial infarction with no obstructive coronary artery disease (MINOCA)) [58]. MINOCA patients are younger, have more NSTEMI events, have lower cardiac troponin levels, and have greater ejection fractions than obstructive MI patients. Females with MINOCA are on average eight years older and have smaller MIs than males. Females also have significantly less multivessel disease than males (30% of females vs. 90% of males), which is consistent with the decreased prevalence of atherosclerotic plaques in females versus males [58]. When in an enhanced systemic inflammatory state (measured by increased vertebral bone marrow activity as a surrogate for leukocyte production) females have decreased myocardial perfusion and decreased left ventricular systolic function whereas no such decrease is seen in males [59].

Myocardial microvasculature considerations

For both males and females, increased myocardial microvasculature resistance is a sensitive predictor of future MI, the severity of a current MI, and adverse cardiac events following MI [60-63]. The two major ways to assess myocardial microvasculature resistance are by using coronary flow reserve (CFR) and an index of microvascular resistance (IMR). CFR is determined by resting mean transit time divided by hyperemic mean transit time of saline across an investigated vessel. IMR is determined by mean distal coronary pressure multiplied by hyperemic mean transit time of saline. Worse scores of either CFR or IMR predict worse outcomes of MI, and worse scores of both of these parameters in the same patient are most predictive of worse outcomes [64]. In MI patients, when examining arteries not responsible for an infarct compared to stable angina patients, MI patients have worse CFR throughout the heart, not just in the arteries involved with the infarcts [65]. Insulin resistance is also predictive of impaired CFR and overall impaired coronary microcirculatory function, again highlighting its important role in the hearts of females and males [66].

A sex-related difference attributable to microvascular differences is unclear. While it is true that small vessel disease is associated with unrecognized MI and a larger proportion of females than males with MI have unrecognized MI, no difference between females and males in microvasculature disease has been repeatedly shown throughout the literature, with few studies examining sex difference [67]. It has been shown that increasing age and time since menopause both are correlated with an overall decrease in systemic capillary refill time. Females who had used oral contraceptives in the past or used hormone replacement therapy after menopause had better microcirculatory function than those who did not, highlighting a possible protective role of both estrogen and progesterone in microcirculatory function and therefore cardiac events [68].

Pain tolerance as a possible differential factor

It has been proposed that sex differences in pain sensitivity may play into symptom differences in MI [18]. There are differences in pain sensitivity between the sexes and in states of inflammation females have a further reduced sensitivity [69]. However, pain tolerance as an explanation for differences in presentation between females and males has only been directly studied to our knowledge one time, where the presence of unrecognized MI was correlated with a higher pain threshold in females but not in males [18].

Type 1 versus type 2 MI

The vast majority of MIs are considered type 1 or type 2. Type 1 are infarcts related to a primary coronary event such as plaque erosion or rupture and type 2 are infarcts related to ischemia or other oxygen supply versus demand mismatches [70,71]. Depending on the study, 15-25% of MIs are type 2, though inconsistent awareness and diagnosis make the true number difficult to ascertain [71-73]. Patients with type 2 are more likely to be older and be females [71-73]. Independent of age, type 2 MIs are more likely to have other comorbidities including (in order of frequency) chronic kidney disease, atrial fibrillation, heart failure, anemia, depression, chronic obstructive pulmonary disease, valvular heart disease, liver disease, alcohol use disorder, and substance abuse disorder [71-73]. Type 1 patients are more likely to have risk factors for plaque formation including (in order of frequency) dyslipidemia, being a smoker, prior percutaneous coronary intervention, prior MI, and prior coronary artery bypass graft [71]. While type 2 patients are more likely to be older females, about 12% of younger females do not fit into one of the classical MI categories as their MIs oftentimes do not have evidence for myocardial supply versus demand mismatch. These categories may need to be amended to include obstructive and nonobstructive coronary artery disease without evidence for supply-demand mismatch to include these patients [74].

Discussion

The objective of this review was to examine the symptomatic and pathophysiological differences between MI in males and females and possible links between the two. Though there are clear differences in both symptoms and pathophysiology of MI in males and females, links between the two are tenuous or omitted altogether from many articles. Symptomatically, females present most often with chest pain when having an MI, but so often present with “atypical” symptoms that the term atypical itself may need to be amended, as females present more often with an atypical symptom than they do without one. Prodromal symptoms also seem to be more common in females, which may be related in part to their increased likelihood of nonobstructive MI. This follows since prodromal symptoms should not occur weeks and months prior to an acute thrombus formation. Risk factor differences between the sexes also exist, with smoking, hypertension, and DM all impacting females more negatively than males. Clear pathophysiological differences also exist between the sexes, with females expressing a lower overall atherosclerotic burden on average than males when they have an MI. This difference in plaque burden, however, has not been evaluated for differences in symptoms in these patients, so its role in symptom differences is unclear at this time. Differences between females and males in the prevalence of type 1 versus type 2 MI also exist, though no studies included in this review examined symptom differences between the two MI variants.

There are a handful of explanations explored in this review for a pathophysiological difference leading to the symptomatic differences between males and females. It has been proposed that the difference in microvasculature resistance between males and females is etiologic for their presentation with atypical symptoms [75]. Increased microvascular resistance has been linked to unrecognized MI in females, and females are more likely to experience greater rates of resistance in their coronary microvasculature following menopause. It may be that these microvascular changes with age and menopause predispose females to more atypical symptoms that then go unrecognized. While no clear-cut causal relationship has been shown, it is a promising area of study for the future that may yield new avenues for MI recognition and treatment in females. It has also been proposed that sex differences in pain tolerance or sensation may predispose females to the wider variety of symptoms that are seen. Only one study has been completed in the area, however, where a link was drawn between unrecognized MI in females and a higher pain threshold. This is an area of research that should be expanded upon, as identifying females with higher pain thresholds may be a key player in the early recognition of symptoms for these patients.

Study limitations

Much of the limitation in this study comes down to a lack of evidence and a lack of studies examining the roles of different pathophysiological states and symptoms. While both symptom differences between males and females and the pathophysiology of MI in males and females have been studied extensively, very few conclusions other than speculation can be drawn about the aspects of physiology and pathophysiology that are causal in these symptom differences between the sexes.

## Conclusions

There are differences between both symptoms and pathophysiology in MIs in females and males. However, few studies have examined a link between the two. Evaluation of symptom changes for patients with differing microvascular resistance and pain tolerance is especially promising in this realm. Differences in symptoms for patients with differing atherosclerotic burden and for patients with type 1 versus type 2 MI have not been studied to our knowledge and both may yield a further explanation of these symptom differences between females and males. Further studies with a focus on pathophysiological or physiological causes of symptom differences in MI between females and males are warranted.
